# Occurrence and Accumulation of Human Enteric Viruses and Phages in Process Water from the Fresh Produce Industry

**DOI:** 10.3390/foods10081853

**Published:** 2021-08-11

**Authors:** Enric Cuevas-Ferrando, Ana Allende, Alba Pérez-Cataluña, Pilar Truchado, Natalia Hernández, Maria Isabel Gil, Gloria Sánchez

**Affiliations:** 1 Department of Preservation and Food Safety Technologies, Institute of Agrochemistry and Food Technology, IATA-CSIC, Av. Agustín Escardino 7, 46980 Valencia, Spain; enric.cuevas@iata.csic.es (E.C.-F.); alba.perez@iata.csic.es (A.P.-C.); 2 Research Group on Microbiology and Quality of Fruits and Vegetables, Department of Food Science and Technology, CEBAS-CSIC, Campus Universitario de Espinardo, 30100 Murcia, Spain; aallende@cebas.csic.es (A.A.); ptruchado@cebas.csic.es (P.T.); nhacosta@cebas.csic.es (N.H.); migil@cebas.csic.es (M.I.G.)

**Keywords:** human enteric viruses, viral indicator, bacteriophages, molecular methods, infectivity, produce, wash water, food safety

## Abstract

The virological quality of process water (PW) used by the produce industry has received limited attention. As a first step to overcoming technical limitations in monitoring viruses in PW, the analytical performance of ultrafiltration was assessed to concentrate viral particles from 20 L of spiked PW. The selected method used for sample concentration of PW was carefully validated, thus enabling the accurate quantification and estimation of viral titers of human enteric viruses and phages. PW from the produce industry was collected periodically from the washing tanks of commercial facilities. The analysis of coliphages was performed by plaque assay, while the occurrence of enteric viruses and crAssphage was determined by molecular techniques. Significant differences in the physicochemical composition of PW, mostly due to the different nature of fresh produce types and differences in the sanitizer used in commercial operation, were observed. Accumulation of crAssphage and coliphages was observed in PW, but correlation with human enteric viruses was not possible due to the low prevalence of these pathogens in the PW analyzed. The obtained results showed that depending on the type of product washed, the product/water ratio and the residual concentrations of the sanitizers, the prevalence and concentration of bacteriophages changed significantly.

## 1. Introduction

Despite being one of the major causes of foodborne outbreaks in high-income countries, human enteric viruses have received comparatively less attention than foodborne pathogenic bacteria. Enteric viruses are the most common etiologic agents identified in produce-associated outbreaks (54%), frequently linked with food-handling issues [1]. The presence of human enteric viruses in irrigation waters has been extensively reported [2,3,4,5,6]. Among others, the viruses most commonly detected in irrigation waters include human norovirus, astrovirus (HAstV), rotavirus A (RV), and hepatitis A virus (HAV) [7]. Moreover, the USA included norovirus in a list of water contaminants that must be regulated in drinking water [8]. Physical and chemical parameters, together with classical microbial indicators such as fecal indicator bacteria (FIB), including fecal coliforms, *Escherichia coli* (*E. coli*), and enterococci, have been widely used to assess the quality of process water (PW) used in different postharvest unit operations. However, the presence of human enteric viruses has not been fully implemented for this purpose. 

Process water has been defined as water resulting from washing raw materials, rinsing water, or water used for cooling or transport, which usually accumulates organic matter, including micro-organisms [9]. Process water in the fruit and vegetable sector is highly variable in terms of quality parameters, such as dissolved solids, chemical oxygen demand, and microbiological quality. This fact makes it a challenge to implement a standard treatment fit for all purposes [10]. The occurrence of potentially infectious enteric viruses in PW used by the fresh produce industry is likely possible, and thus, it must be closely examined. Several factors must be considered to address this issue: (i) Relatively low levels of enteric viruses introduced will be randomly distributed into large volumes of water and may not be detectable using protocols indicating small volume collection; (ii) sampling points in commercial facilities are critical for pathogen detection [11]; (iii) molecular-based methods, currently used for enteric virus detection in food [12] cannot discriminate between inactivated and potentially infectious enteric viruses; (iv) organic fresh produce market, limiting the use of sanitizers, has tremendously increased in the last years and the food safety perception of consumers must be assured. 

Due to the difficulties associated with direct detection of viral pathogens in water, bacteriophages infecting enteric bacteria, such as coliphages, have been suggested as a viral indicator in irrigation water because they mimic viruses better than any other group of indicators showing moderate resistance to treatments and persistence in the environment [13]. Coliphages are viruses used as viral indicators and can infect *E. coli.* They are split into two categories based on the route of bacterial host infection: somatic coliphages and male-specific (F+) coliphages (F-RNA & F-DNA) [14]. Recently, crAssphage (cross-assembly phage) has been suggested as a novel viral indicator of fecal contamination as it is present in high abundance compared with human enteric viruses. Recent data indicate that crAssphage can be used to detect human fecal contamination on environmental surfaces and hands [15]. However, the usefulness of this indicator in PW is still unknown.

Monitoring and maintaining the quality of PW during postharvest operations is considered important for both the safety and quality of end-products [10]. In this study, we monitored the occurrence of the most relevant human enteric viruses, coliphages, and crAssphage in PW collected from three different processing plants of whole and fresh-cut fruits and vegetables during six sampling times from July to December 2020. To monitor PW, a rapid, user-friendly, and reliable protocol to concentrate human enteric viruses, as well as bacteriophages in large PW volumes, was initially developed. Importantly, limits of detection were established using model human enteric viruses and MS2 phage. 

## 2. Materials and Methods

### 2.1. Viruses, Phages, Cells and Bacteria

Feces positive for norovirus GI, norovirus GII, and HAstV (courtesy of Dr. Buesa from Hospital Clínico Universitario, University of Valencia, Valencia, Spain) were resuspended (10%, *wt*/*vol*) in phosphate-buffered saline (PBS) containing 2 M NaNO_3_ (Panreac, Barcelona, Spain), 1% beef extract (Laboratorios Conda, Madrid, Spain), and 0.1% Triton X-100 (Fisher Scientific, Madrid, Spain) (pH 7.2), vortexed and centrifuged at 1000× *g* for 5 min. The supernatant was stored at −80 °C in aliquots. Mengovirus was used as a process control for sample concentration validation [16]. The cytopathogenic HM-175 strain of HAV (ATCC VR-1402) and the human RV strain Wa (ATCC VR-2018, American Type Culture Collection, Rockville, MD, USA), and mengovirus vMC0 (CECT 100000, Spanish Type Culture Collection, Valencia, Spain) were propagated in FRhK, MA-104, and HeLa cell monolayers, respectively. Semipurified stocks were produced afterward in the same cells by low-speed centrifugations of infected cell lysates (3000× *g* for 20 min). 

The *E. coli* strains CECT 9198, and CECT 5695 were obtained from the Spanish Type Culture Collection and used to quantify total and F-specific RNA phages, respectively. Wild-type MS2 bacteriophage DSM 13767 was obtained from the German Collection of Microorganisms and Cell Cultures.

### 2.2. Physicochemical Properties of Process Wash Water

The pH, oxidation-reduction potential (ORP), and electric conductivity (EC, µS/cm) were measured using a pH and redox multimeter (Crison, Barcelona, Spain). Organic matter was measured as chemical oxygen demand (COD) determined by the standard photometric method [17] using the Spectroquant NOVA 60 photometer. Turbidity was tested using the Turbiquant 3000R turbidimeter (Merck, Madrid, Spain).

Sanitizer concentrations were determined using Kemio^®^ (Palintest, Gateshead, UK), based on a chronoamperometry measurement with the corresponding sensors for chlorine and peracetic acid (PAA). For quenching the disinfectant residuals, sodium thiosulfate (Panreac, Castellar del Vallés, Spain) was used for chlorine, and a mix of sodium thiosulphate and catalase was prepared in phosphate buffer (Merck, Darmstadt, Germany) for PAA.

### 2.3. Concentration Procedure

Process water from washing shredded lettuce was generated in the laboratory, mimicking the industrial conditions described previously [18]. Briefly, to generate the PW, lettuce heads were obtained from a local supermarket (Murcia, Spain), cut into 6 mm pieces after the outer leaves were manually removed, and washed in tap water. A total of 6 kg of cut lettuce was added to 8 L of tap water to obtain PW with similar physicochemical characteristics to those obtained in commercial processing lines. PW was then artificially inoculated with the MS2 bacteriophage (10^6^ plaque-forming units PFU/mL), a single-stranded RNA virus, and mengovirus to evaluate the performance of virus concentration methods. For the primary concentration of viruses, 20 L of process water was processed by dead-end hollow fiber ultrafiltration (DEUF) using single-use Asahi Kasei Rexeed-25A filters [19]. In order to recover the viruses, the filter was backflushed using 500 mL of backflush solution (0.01% Tween 80, 0.01% sodium polyphosphate, and 0.001% antifoam). The backflush volume was concentrated using polyethylene glycol (PEG) precipitation (100 g/L), and the final concentrate was used to extract viral RNA. Mengovirus was then quantified by RT-qPCR according to ISO 15216:2017, and MS2 was enumerated using the host strain *E. coli* CECT 9198 (Spanish Type Culture Collection) and the double-layer agar method. Plaque count assays for MS2 enumeration were performed in Luria–Bertani agar incubated at 37 °C for 24 h. In parallel, 1 L of artificially inoculated PW was concentrated using an aluminum hydroxide adsorption-precipitation method [16].

### 2.4. Detection Limit of Enteric Viruses and MS2 in Process Water

Process water (20 L) from a leafy green line was collected 4–5 h after the process started and transported to the lab. Once in the lab, PW was characterized as previously mentioned. Free chlorine (FC) and total chlorine (TC) levels were determined by the DPD method using the Spectroquant NOVA 60 photometer (Merck, Darmstadt, Germany) and the corresponding test kits [17]. Combined chlorine (CC) values were calculated by differences in the measurements between TC and FC. In the case of residual concentrations of disinfectants present in the PW, as previously mentioned, a solution of sodium thiosulphate pentahydrate (Scharlau, Barcelona, Spain) was used for quenching the disinfectant residuals. Process water was then artificially inoculated with different serial ten-fold diluted concentrations (starting from 6 log_10_ IU/20 L) of norovirus genogroup (GI), norovirus GII, and rotavirus, establishing the detection limit and recoveries of the procedures. In addition, mengovirus was used as the process control. Primary virus concentration was performed using DEUF with Rexeed-25A filters.

### 2.5. Viral Extraction, Detection and Quantification

Nucleic acids from each concentrated PW were extracted following the NucleoSpin^®^ RNA virus kit (Macherey–Nagel GmbH & Co., Düren, Germany) manufacturer’s instructions with some modifications. In short, 150 µL of each concentrated sample was added to 25 μL Plant RNA Isolation Aid (Ambion, Carlsbad, CA, USA), 600 μL of lysis buffer from the NucleoSpin^®^ RNA virus kit and subjected to pulse-vortexing. The homogenate was then centrifuged for 5 min at 10,000× *g* for debris removal. The supernatant was subsequently processed according to the manufacturer’s instructions. The presence of norovirus GI and GII, HAV, HAstV, RV, and mengovirus was detected in 96-well plates (Axygen, Reynosa, MexicoSpain) using the RNA UltraSense One-Step kit (Invitrogen, Frederick, MA, USA), whilst crAssphage occurrence was resolved through qPCR Premix Ex Taq™ kit (Takara Bio Inc., Kusatsu, Japan). For both RT-qPCR and qPCR assays, LightCycler^®^ 480 instrument (Roche Diagnostics, Mannheim, Germany) was used for amplification and detection analysis. Moreover, undiluted and ten-fold diluted nucleic acid were tested to check for inhibitors.

Different controls were used in all assays, including negative process control consisting of PBS, whole process control to monitor the process efficiency of each sample (spiked mengovirus), and positive and negative RT-qPCR controls. Primers, probes, and RT-qPCR conditions used in this study are listed in Table S1.

Standard curves were determined according to the Public Health England (PHE) Reference Materials for Microbiology for norovirus GI (batch number 0122-17), norovirus GII (batch number 0247-17), HAV (batch number 0261-2017) and reported as genomic copies (GC), while standard curves for RV, mengovirus, and HAstV were generated by amplifying ten-fold serial dilutions of viral suspensions in quintuplicates and calculating the number of PCR units (PCRU). Standard DNA material for crAssphage standard curve generation relied on a customized gBlock gene fragment containing target sequences for crAssphage (Integrated DNA Technologies, Coralville, IA, USA) [20,21].

### 2.6. Types of Process Water Analyzed

Three industrial collaborators, including vegetable processors of baby leaves, veggie fruit mixes, and a handler and packer of sweet peppers, were visited individually six times from July to December 2020. Chlorine was used as a sanitizer for baby leaves, while PAA was used for washing peppers. No sanitizer was used either for the prewashing of peppers or the washing of veggie fruit mix. A volume of 20 L of PW from the washing tanks used for the prewashing and washing was collected 4–5 h after production started and transported to the lab in less than 45 min. Once in the lab, PW was characterized as previously described. Water samples from three processing plants (baby leaves, peppers, and veggie fruit mix) were concentrated and analyzed as previously described for coliphages, human enteric virus, and crAssphage detection and quantification. 

## 3. Results and Discussion

### 3.1. Optimization of Concentration Procedure of Process Water for Enteric Virus Detection

The surveillance of PW for the presence of enteric viruses requires procedures sensitive enough to detect the low levels of viruses expected. Initially, two different approaches previously used for human enteric virus detection in wastewaters were compared using PW artificially generated (Artificial PW) and PW from the industry (Industrial PW). The physicochemical characteristics of both PWs are listed in Table 1. The mean recoveries of mengovirus in PW ranged from 23.73 ± 0.21% in the DEUF procedure using 20 L of PW to 23.56 ± 0.53% in aluminum precipitation using 1 L of PW, which are slightly higher than the mengovirus recovery rates using Rexeed 25AX ultrafiltration reported previously in tap water, seawater, and surface water [19,22]. In the case of MS2, the mean recoveries were 31.72 ± 11.47 and 47.91 ± 19.50 for DEUF and aluminum precipitation, respectively. In light of those results, the DEUF protocol was selected to determine the detection limit due to the higher volume being processed (20 L versus 1 L); this is a tremendous advantage in samples expected to contain low concentrations of pathogens, as well as recover viable phages.

### 3.2. Determination of the Detection Limit in PW for Enteric Viruses and MS2

One major limitation in determining the virological quality of PW is the lack of standardized and validated methods. Thus, the detection limits of human enteric viruses and MS2 in PW were examined by analyzing serial diluted spiked samples (Tables S2 and S3). Primary virus concentration was performed using DEUF with Rexeed-25A filters, resulting in an average eluate volume of 609.25 ± 60.40 mL. The DEUF ultrafiltration combined with secondary PEG precipitation resulted in the mean recovery of 27.10%, 27.31%, 36.56%, and 39.03% for norovirus GI, GII, rotavirus, and MS2. An average recovery rate of 19% for mengovirus was achieved, which meets the quality control requirements of standardized methods such as the ISO 15216-1 (requires 1% recovery of the process control) or the Method 1615 from the US Environmental Protection Agency (EPA) (allows 5–200% recoveries for the process control) for the concentration of environmental and drinking water samples for the quantification of enteric viruses. According to Wilrich and Wilrich (2009), the LoD95% of the procedure in PW was calculated as 1.09 × 10^3^ gc/L, 1.71 × 10^3^ gc/L, 4.28 × 10^2^ gc/L and 2.62 × 10^2^ pfu/L for norovirus GI, GII, rotavirus, and MS2, reporting similar performance, 6.2 × 10^3^ gc/L, when the method was applied for HEV in drinking [23,24]. Farkas et al. (2018) reported LoD of 50 gc/L for norovirus using a similar procedure; however, validation was performed using deionized water only [25].

### 3.3. Occurrence of Coliphages and Human Enteric Viruses in Process Water

Three commercial processing plants, including two vegetable processors of baby leaves, veggie fruit mix, and a handler and packer of sweet peppers, were visited individually six times from July to December 2020. The physicochemical characteristics of the PWs are shown in Table S4. 

For baby leaves, the mean ratio of produce/ wash water was 1.6 kg/L of water. The concentration of FC was maintained between 0–59 and 3–125 mg/L in the prewashing and washing tanks, respectively. The pH of the chlorinated water was 8.5, higher than recommended (6.5), to reach the maximum concentration of hypochlorous acid when sodium hypochlorite is used as a water disinfectant; this means that the residual concentration of free chlorine with the maximum antimicrobial capacity would be lower than that expected at the optimum pH. The ORP was higher than 650 mV, indicating reactive oxidizing species, except in one sampling where no FC was present and the ORP was lower (463 mV). The content of organic matter was low (188 and 75 mg/L maximum in the prewashing and washing tanks, respectively), as well as UV254 absorbance and turbidity. When the levels of total and F-specific RNA coliphages were determined in prewash and washing PW of baby leaves, it was observed that no recovery of either total or F-specific RNA coliphages in any of the prewash or the washing tanks occurred. However, detection of crAssphage was observed in 50% of the samples (Table 2). The absence of viable phages in these samples indicates that detection of crAssphage by PCR was most likely targeting crAssphage DNA traces rather than viable phages. Furthermore, the absence of viable phages was probably due to the residual FC level even though the pH was higher than recommended. During the surveillance of human enteric viruses in PW from baby leaves after six months of testing, no human enteric viruses were found; therefore, correlations with phages, either detected by culture or PCR, could not be established. Inactivation studies using PW from the produce industry reported 3-log reduction for murine norovirus (MNV) and MS2 viability at 0.2 and 2.5 mg residual FC min/L, respectively [26]. These sanitizer concentrations are much lower than the doses currently applied by the produce industry.

For bell peppers, the mean ratio of produce/wash water was 71 kg/L of water, which is an extraordinarily low volume of water compared with a considerably high amount of product (Table S4). This fact influenced the physicochemical quality of the prewashing water with no PAA and the washing water with PAA. The peracetic acid concentration was very high, with mean values of 334 mg/L vs. 80 mg/L, the recommended one. We observed a very high COD with a mean value of 455 and 1490 mg/L in the prewashing and washing tanks, respectively. In the prewashing PW, the EC was 743 µS cm^−1^ and the turbidity was 392 NTU. In the washing PW, the EC was 765 µS cm^−1^ and the turbidity was 159 NTU. These physicochemical characteristics influenced the UV254 absorbance, which showed high levels (1.4 Abs) and low redox potential. The ORP when using PAA is generally lower than 650 mV, which is typically found in chlorinated water (>650 mV). It is remarkable to mention the high turbidity of the prewashing PW due to the high amount of bell peppers washed in a small volume of water without any water replenishment and the consequent accumulation of organic and inorganic residues from the product. High levels of coliphages, total and F-specific RNA, of about 4-log pfu/L were found in the prewashing tank (Table 2). The high levels of coliphages in the prewashing water were probably due to the absence of sanitizer and the high ratio of produce/water. However, when a high residual concentration of PAA was maintained in the washing tank, the counts of total and F-specific RNA coliphages decreased one log with respect to the prewashing counts, with no differences between total and F-specific RNA coliphages (Table 2). Previous studies reported for MNV, a norovirus surrogate, that lower doses of PAA (40, 80, and 120 ppm) could inactivate about 3.8 log MNV in artificially inoculated PW from the strawberry industry [27]. However, the conditions used in the mentioned study were very different from those of the industry.

The veggie fruit mix included tomatoes, peppers, cucumbers, and onions, which all entered the washing line before the blended process. The main difference of this PW was that no sanitizer was added. The physicochemical characteristics of the PW showed that the quality was very satisfactory because of the low COD value (166 mg/L), the pH was close to neutral (mean of 7.3), the ORP was very low (mean of 218 mV), as well as the UV254 (0.08), and turbidity (19 NTU). Counts of total and F-specific RNA coliphages varied considerably in the samples from non-detected coliphages to high counts (4.1 log pfu/L) with no significant differences between them (Table 2). These high levels of coliphages were probably due to the absence of sanitizer in the water tank (Table 2).

The presence of human enteric viruses in vegetables has been reported with markedly varying levels between studies [28,29,30,31,32]. Viral contamination that may be present on the product can spread throughout the production batch when the product is washed, warranting investigation about the levels of human enteric viruses in PW. In this study, the majority of the samples tested negative for the presence of human norovirus GI, GII, astrovirus, rotavirus, and HAV, except in one PW sample from peppers, in which rotavirus was detected. Overall, detection of crAssphage was observed in 70 and 60% of samples from the prewashing and washing tanks, respectively; this is the first time that this bacteriophage has been detected in PW. Correlation between the prevalence and concentration of crAssphage and enteric viruses was explored. However, among all the samples that tested positive for crAssphage, only one tested positive for enteric viruses. Therefore, based on the observed data, crAssphage cannot be suggested as a good indicator for the presence of enteric viruses in PW. Recent studies have shown a correlation between crAssphage and human viral pathogens in other water matrices such as wastewaters [33,34,35,36,37,38], sludge [39], and other fecal polluted waters [40]. Altogether, these and our results suggest that crAssphage correlates with the occurrence of human pathogenic enteric viruses in water samples with moderate viral contamination (e.g., effluent waters), but not in severely or poorly contaminated waters (e.g., surface, river, influent, seawater, or process water).

## 4. Conclusions

Process water obtained from three different commercially handled and processed lines of fruits and vegetables showed significant differences in their physicochemical composition primarily due to the diverse nature of the product type and the use or non-use of sanitizers (chlorine and PAA). The recoveries and LoD achieved with the method optimized for PW suggested that this procedure can be standardized and used for routine monitoring. This method was suitable for detecting and quantifying (RT)-qPCR of different types of viruses, including enteric viruses and crAssphage, as well as viable bacteriophages, including total coliphages, F-specific RNA phages, and MS2. The obtained results showed that depending on the product, the water ratio, the type of product washed in the water, and the residual concentration of the sanitizer, the prevalence, and concentration of bacteriophages varied significantly. The concentration of coliphages and crAssphage was the highest in PW with a low replenishment rate and no sanitizers. On the contrary, the prevalence and concentrations of bacteriophages were much lower when residual chlorine was constantly maintained. An intermediate situation was illustrated in washing peppers as phages accumulated in PW even though the prevalence of the enteric viruses was very low. Based on the limit of detection for enteric viruses, it may be possible that the viruses were present, but the method’s sensitivity was not adequate for their detection and quantification. More research should be done to lower the detection limit to confirm the low potential risk linked to the accumulation of enteric viruses in PW when a residual sanitizer is present. Our results cannot suggest the use of crAssphage as a good indicator of human enteric viruses in PW, mainly because of the low prevalence of viruses in the present study.

## Figures and Tables

**Table 1:** Physicochemical characterization of PW.

Type of Water	pH	ORP	COD	EC
Artificial PW	5.8 ± 0.5	374.5 ± 4.2	892.3 ± 53.5	733.2 ± 104.3
Industrial PW	7.8 ± 0.1	279.8 ± 14.2	1429.8 ± 184.4	955.5 ± 317.7

Abbreviations: ORP: oxidation reduction potential (m/V); COD: chemical oxygen demand (mg/L); EC: electric conductivity (µS cm^−1^).

**Table 2:** Levels of total and F-specific RNA coliphages, crAssphage, and human enteric viruses (norovirus GI and GII, HAV, HAstV, RV) of wash water from the prewashing and washing steps of baby leaves, bell pepper, and veggie fruit mix of commercial lines using sodium hypochlorite, peracetic acid (PAA) and without sanitizer, respectively.

Produce	Sampled Water	Sampling Date	Sanitizer	Sanitizer Concentration (mg/L)	Total Coliphages ^1^	F-Specific RNA Coliphages ^1^	crAssphage ^1^	Human Enteric Viruses ^1^
Baby leaves	Prewashing	21 July 2020	Chlorine	18 ± 1	Nd	Nd	Nd	Nd
		30 September 2020	Chlorine	0 ± 0	Nd	Nd	2.18 ± 0.00	Nd
		13 October 2020	Chlorine	36 ± 1	Nd	Nd	Nd	Nd
		27 October 2020	Chlorine	37 ± 2	Nd	Nd	Nd	Nd
		10 November 2020	Chlorine	57 ± 6	Nd	Nd	2.97 ± 0.06	Nd
		24 November 2020	Chlorine	59 ± 0	Nd	Nd	2.46 ± 0.04	Nd
	Washing	21 July 2020	Chlorine	12 ± 1	Nd	Nd	Nd	Nd
		30 September 2020	Chlorine	3 ± 0	Nd	Nd	Nd	Nd
		13 October 2020	Chlorine	98 ± 8	Nd	Nd	Nd	Nd
		27 October 2020	Chlorine	72 ± 1	Nd	Nd	1.66 ± 0.00	Nd
		10 November 2020	Chlorine	120 ± 3	Nd	Nd	2.93 ± 0.04	Nd
		24 November 2020	Chlorine	125 ± 5	Nd	Nd	2.62 ± 0.14	Nd
Bell peppers	Prewashing	21 July 2020	None	-	4.4 ± 0.0	4.4 ± 0.0	Nd	Nd
		29 September 2020	None	-	3.4 ± 0.0	3.1 ± 0.0	3.08 ± 0.02	Nd
		13 October 2020	None	-	3.8 ± 0.1	3.9 ± 0.0	1.18 ± 0.00	Nd
		27 October 2020	None	-	3.6 ± 0.1	3.9 ± 0.0	Nd	Nd
		10 November 2020	None	-	4.3 ± 0.0	4.0 ± 0.0	3.02 ± 0.04	Nd
		24 November 2020	None	-	3.5 ± 0.0	3.3 ± 0.0	3.14 ± 0.20	Nd
	Washing	21 July 2020	PAA	416 ± 96	4.4 ± 0.0	4.4 ± 0.0	Nd	Nd
		29 September 2020	PAA	233 ± 2	Nd	Nd	Nd	Nd
		13 October 2020	PAA	326 ± 9	2.9 ± 0.0	3.0 ± 0.0	1.18 ± 0.00	Nd
		27 October 2020	PAA	330 ± 7	3.3 ± 0.1	3.8 ± 0.0	Nd	Nd
		10 November 2020	PAA	370 ± 9	3.7 ± 0.2	3.6 ± 0.0	Nd	Nd
		24 November 2020	PAA	332 ± 9	2.7 ± 0.1	3.1 ± 0.1	2.94 ± 0.09	5.55 *
Veggie fruit mix	Washing	29 September 2020	None	-	3.9 ± 0.1	4.1 ± 0.0	Nd	Nd
		14 October 2020	None	-	2.0 ± 0.0	2.0 ± 0.0	Nd	Nd
		27 October 2020	None	-	Nd	Nd	2.11 ± 0.08	Nd
		24 November 2020	None	-	3.8 ± 0.1	3.7 ± 0.0	Nd	Nd
		9 December 2020	None	-	3.7 ± 0.1	3.6 ± 0.1	2.82 ± 0.04	Nd
		22 December 2020	None	-	2.7 ± 0.1	2.0 ± 0.0	2.33 ± 0.21	Nd

^1^ Bacteriophage load expressed as log pfu/L and viral load as log gc/L. Nd, Not detected. *, only rotavirus detection. “-“, No sanitizer added.

## Data Availability

The datasets generated for this study are available on request to the corresponding author.

## Supplementary Materials

The following are available online at https://www.mdpi.com/article/10.3390/foods10081853/s1, Table S1: Primers, probes, and (RT)-qPCR conditions used in the study. Table S2: Detection limit of enteric viruses in process water. Table S3: Limit of detection of MS2 in process water by Rexeed 25AX ultrafiltration followed by precipitation with polyethylene glycol using two *Escherichia coli* strains. Table S4: Physicochemical characteristics including sampling dates, ratio product/water, chemical oxygen demand (COD), pH, oxidation-reduction potential (ORP), absorbance at 254 nm (UV254), turbidity, electrical conductivity (EC), and temperature (°C) of process water from the prewashing and washing steps of baby leaves, bell pepper, and veggie fruit mix from commercial lines using sodium hypochlorite, peracetic acid (PAA) and without sanitizer, respectively. Ref. [41] is cited in Supplementary Materials.
