# Supplementary material for: Occurrence and Accumulation of Human Enteric Viruses and Phages in Process Water from the Fresh Produce Industry
Source: Foods. 2021 Aug 11;10(8):1853. doi: 10.3390/foods10081853 (PMC8391481; doi:10.3390/foods10081853)
Supplement: Supplementary file 1 [file foods-10-01853-s001.zip › foods-1318428-supplementary.pdf]

Supplementary material

Table S1. Primers, probes and (RT)-qPCR conditions used in the study.

Virus	Primers and probe	Sequence	RT-qPCR conditions	Reference
Norovirus GI	QNIF4	CGC TGG ATG CGN TTC CAT	RT: 55 °C for 60 min, Preheating: 95 °C for 5 min PCR (45 cycles) 95 °C for 15 s, 60 °C for 60 s, 65 °C for 60 s.	[1]
	NV1LCR	CCT TAG ACG CCA TCA TCA TTT AC		
	NVGG1p	TGG ACA GGA GAY CGC RAT CT		
Norovirus GII	QNIF2	ATG TTC AGR TGG ATG AGR TTC TCW GA	RT: 55 °C for 60 min, Preheating: 95 °C for 5 min PCR (45 cycles) 95 °C for 15 s, 60 °C for 60 s, 65 °C for 60 s.	[1]
	COG2R	TCG ACG CCA TCT TCA TTC ACA		
	QNIFs	AGC ACG TGG GAG GGC GAT CG		
HAV	HAV68	TCA CCG CCG TTT GCC TAG	RT: 55 °C for 60 min, Preheating: 95 °C for 5 min PCR (45 cycles) 95 °C for 15 s, 60 °C for 60 s, 65 °C for 60 s.	[1]
	HAV240	GGA GAG CCC TGG AAG AAA G		
	HAV150	CCT GAA CCT GCA GGA ATT AA		
RV	JVKF	CAG TGG TTG ATG CTC AAG ATG GA	RT: 50 °C for 30 min, Preheating: 95 °C for 15 min PCR (45 cycles) 94 °C for 10 s, 55 °C for 30 s, 72 °C for 20 s.	[2]
	JVKR	TCA TTG TAA TCA TAT TGA ATA CCC A		
	JVKP	FAM-ACA ACT GCA GCT TCA AAA GAA GWG T		
HAstV	AstVorf1b+	AAG CAG CTT CGT GAC TCT GG	RT: 55 °C for 60 min, Preheating: 95 °C for 5 min PCR (45 cycles) 95 °C for 15 s, 58 °C for 60 s, 65 °C for 60 s.	[1]
	AstVorf1b-	AGC CAT CAC ACT TCT TTG GTC		
	AstVorf1bp			
MgV	Mengo 110	GCG GGT CCT GCC GAA AGT	RT: 55 °C for 60 min, Preheating: 95 °C for 5 min PCR (45 cycles) 95 °C for 15 s, 60 °C for 60 s, 65 °C for 60 s.	[1]
	Mengo 209	GAA GTA ACA TAT AGA CAG ACG CAC AC		
	Mengo 147	ATC ACA TTA CTG GCC GAA GC		
crAssphage	064F1	TGT ATA GAT GCT GCT GCA ACT GTA CTC	Preheating: 95 °C for 5 min PCR (45 cycles) 95 °C for 5 s, 60 °C for 30 s,	[3]
	064R	CGT TGT TTT CAT CTT TAT CTT GTC CAT		
	064P1	CTG AAA TTG TTC ATA AGC AA		

Table S2. Detection limit of enteric viruses in process water.

**Virus**	**Inoculated viral titer**					LoD$_{95\%}$ **(gc/L)**	Recovery **(%)**
	≈10^5	≈10^4	≈10^3	≈10^2	≈10		
Norovirus GI	4/4[1]	4/4	4/4	1/4	0/4	1.09×10^3	27.10
Norovirus GII	4/4	4/4	4/4	3/4	0/4	1.71×10^3	27.31
Rotavirus	4/4	4/4	4/4	3/4	0/4	4.28×10^2	36.56

[1] Virus positive/total numbers of samples. Abbreviations: LoD$^{95\%}$: limit of detection (gc/L) calculated according to Wilrich and Wilrich, 2009.

Table S3. Limit of detection of MS2 in process water by Rexeed 25AX ultrafiltration followed by precipitation with polyethylene glycol using two *E. coli* strains.

	**Host**	**≈1 x 10^5**	**Recovery (%)**	**≈1 x 10^4**	**Recovery (%)**	**≈1 x 10^3**	**Recovery (%)**
**Total**	*E. coli* 9198	30450	36.91	2343.25	12.33	306.9	32.14
**F-specific RNA**	*E. coli* 5695	17850	46.36	4377.5	23.04	254.2	69.64

	**Host**	**≈1 x 10^2**	**Recovery (%)**	**≈1 x 10^0**	**Recovery (%) (%)**	**$LoD_{95\%}$ (pfu/L)**
**Total**	*E. coli* 9198	54.25	56.81	0.412	19.15	**4.11 ×10^2**
**F-specific RNA**	*E. coli* 5695	32.20	88.22	0.114	5.69	**1.13 ×10^2**

Table S4. Physicochemical characteristics including pH, oxidation-reduction potential (ORP), absorbance at 254 nm (UV254), turbidity, chemical oxygen demand (COD), electrical conductivity (EC), and temperature of wash water from the pre-washing and washing steps of baby leaves, bell pepper and veggie fruit mix of commercial lines using sodium hypochlorite, peracetic acid (PAA) and without sanitizer, respectively. NA: Not Available. Values are the mean ± standard deviation (n = 3).

Sampled water		Date	Ratio Produce/water (kg/L)	COD	pH	ORP (mV)	UV254 (Abs.)	Turbidity (NTU)	EC (µS cm^{-1})	Temperature (ºC)
Baby leaves	Pre-Washing	7/21/2020	1.6	188 ± 39	7.9 ± 0.1	705 ± 13	0.16 ± 0.01	172 ± 3	918 ± 1	12 ± 0
		9/30/2020	1.2	67 ± 8	7.8 ± 0.1	463 ± 34	0.06 ± 0.00	57 ± 1	781 ± 2	9 ± 0
		10/13/2020	1.7	54 ± 25	8.1 ± 0.0	710 ± 5	0.07 ± 0.00	23 ± 3	866 ± 2	6 ± 0
		10/27/2020	1.8	42 ± 7	8.1 ± 0.1	717 ± 17	0.06 ± 0.00	24 ± 3	1156 ± 2	14 ± 1
		11/10/2020	1.6	32 ± 17	8.5 ± 0.0	755 ± 1	0.09 ± 0.00	45 ± 1	997 ± 2	9 ± 0
		11/24/2020	1.8	71 ± 6	8.3 ± 0.1	738 ±13	0.11 ± 0.01	51 ± 5	1106 ± 2	7 ± 1
	Washing	7/21/2020	1.6	75 ± 35	7.8 ± 0.1	724 ± 6	0.05 ± 0.00	31 ± 1	803 ± 2	11 ± 0
		9/30/2020	1.2	23 ± 3	7.7 ± 0.1	643 ± 12	0.02 ± 0.00	14 ± 1	725 ± 4	10 ± 0
		10/13/2020	1.7	35 ± 3	8.3 ± 0.1	732 ± 3	0.13 ± 0.00	15 ± 1	1108 ± 1	7 ± 0
		10/27/2020	1.8	19 ± 3	8.2 ± 0.0	753 ± 3	0.09 ± 0.00	12 ± 0	1397 ± 2	13 ± 0
		11/10/2020	1.6	14 ± 12	8.5 ± 0.1	748 ± 3	0.15 ± 0.00	21 ± 1	1288 ± 9	10 ± 0
		11/24/2020	1.8	26 ± 16	8.4 ± 0.0	759 ± 3	0.15 ± 0.00	19 ± 1	1341 ± 6	7 ± 0
Bell peppers	Pre-Washing	7/21/2020	31.0	845 ± 106	NA	NA	0.40 ± 0.01	305 ± 15	NA	NA
		9/29/2020	32.0	301 ± 14	7.4 ± 0.1	205 ± 11	0.29 ± 0.00	241 ± 3	783 ± 2	19 ± 0
		10/13/2020	4.0	414 ± 10	7.5 ± 0.3	216 ± 22	0.29 ± 0.01	376 ± 33	718 ± 1	15 ± 0
		10/27/2020	137.7	281 ± 8	8.0 ± 0.1	438 ± 58	0.40 ± 0.01	425 ± 9	713 ± 4	19 ± 0
		11/10/2020	88.0	548 ± 0	6.7 ± 0.4	226 ± 3	0.77 ± 0.02	538 ± 24	761 ± 2	20 ± 0
		11/24/2020	136.0	341 ± 20	6.8 ± 0.5	189 ± 1	0.47 ± 0.00	467 ± 3	742 ± 4	17 ± 0
	Washing	7/21/2020	31.0	1020 ± 35	3.5 ± 0.0	438 ± 3	1.09 ± 0.00	60 ± 1	815 ± 3	21 ± 0
		9/29/2020	32.0	1541 ± 98	4.0 ± 0.0	465 ± 8	0.94 ± 0.07	49 ± 5	711 ± 6	22 ± 0
		10/13/2020	4.0	2118 ± 43	4.0 ± 0.0	513 ± 9	1.25 ± 0.01	92 ± 2	715 ± 2	16 ± 0
		10/27/2020	137.7	1207 ± 46	4.0 ± 0.0	385 ± 0	1.39 ± 0.00	152 ± 4	728 ± 6	20 ± 0
		11/10/2020	88.0	1900 ± 0	3.8 ± 0.0	382 ± 2	1.93 ± 0.01	390 ± 99	836 ± 8	21 ± 0
		11/24/2020	136.0	1192 ± 25	3.7 ± 0.0	380 ± 1	1.58 ± 0.00	209 ± 2	786 ± 4	17 ± 0
Veggie fruit mix	Washing	9/29/2020	5.6	171 ± 8	7.5 ± 0.3	147 ± 9	0.07 ± 0.00	18 ± 0	666 ± 0	20 ± 0
		10/14/2020	18.4	144 ± 2	7.2 ± 0.4	244 ± 19	0.07 ± 0.00	16 ± 1	882 ± 1	19 ± 0
		10/27/2020	2.9	147 ± 8	7.4 ± 0.2	273 ± 5	0.07 ± 0.00	18 ± 2	732 ± 4	19 ± 0
		11/24/2020	9.1	169 ± 16	7.7 ± 0.2	164 ± 14	0.07 ± 0.00	23 ± 2	917 ± 2	14 ± 0
		12/9/2020	7.8	179 ± 6	7.0 ± 0.3	254 ± 12	0.09 ± 0.00	18 ± 0	1059 ± 2	15 ± 1
		12/22/2020	13.1	185 ± 1	7.2 ± 0.4	226 ± 20	0.09 ± 0.00	19 ± 1	1017 ± 1	14 ± 0

References

1. ISO. ISO 15216-1:2017—Microbiology of the Food Chain—Horizontal Method for Determination of Hepatitis A Virus and Norovirus Using Real-Time RT-PCR—Part 1: Method for Quantification. Available online: https://www.iso.org/standard/65681.html (accessed on 2 June 2021).
2. Jothikumar N, Kang G, Hill VR. Broadly reactive TaqMan® assay for real-time RT-PCR detection of rotavirus in clinical and environmental samples. J Virol Methods. Available online: https://pubmed.ncbi.nlm.nih.gov/18951923/ (accessed on 2 June 2021).
3. Stachler E, Kelty C, Sivaganesan M, Li X, Bibby K, Shanks OC. Quantitative CrAssphage PCR Assays for Human Fecal Pollution Measurement. *Environ Sci Technol* **2017**, *51*(16):9146–54.
